# How to spend the summer? Free-living dormice (*Glis glis*) can hibernate for 11 months in non-reproductive years

**DOI:** 10.1007/s00360-015-0929-1

**Published:** 2015-08-21

**Authors:** Franz Hoelzl, Claudia Bieber, Jessica S. Cornils, Hanno Gerritsmann, Gabrielle L. Stalder, Chris Walzer, Thomas Ruf

**Affiliations:** Department of Integrative Biology and Evolution, University of Veterinary Medicine, Vienna, Savoyenstraße 1, 1160 Vienna, Austria

**Keywords:** Summer dormancy, Torpor, Reproduction, Seasonality, Foraging, Body mass

## Abstract

Edible dormice are arboreal rodents adapted to yearly fluctuations in seed production of European beech, a major food source for this species. In years of low beech seed abundance, dormice skip reproduction and non-reproductive dormice fed ad libitum in captivity can display summer dormancy in addition to winter hibernation. To test whether summer dormancy, that is, a very early onset of hibernation, actually occurs in free-living dormice, we monitored core body temperature (*T*_b_) over ~12 months in 17 animals during a year of beech seeding failure in the Vienna Woods. We found that 8 out of 17 dormice indeed re-entered hibernation as early as in June/July, with five of them having extreme hibernation durations of 11 months or more (total range: 7.8–11.4 months). Thus, we show for the first time that a free-living mammal relying on natural food resources can continuously hibernate for >11 months. Early onset of hibernation was associated with high body mass in the spring, but the distribution of hibernation onset was bimodal with prolonged hibernation starting either early (prior to July 28) or late (after August 30). This could not be explained by differences in body mass alone. Animals with a late hibernation onset continued to maintain high nocturnal *T*_b_’s throughout summer but used short, shallow torpor bouts (mean duration 7.44 ± 0.9 h), as well as occasional multiday torpor for up to 161 h.

**Electronic supplementary material:**

The online version of this article (doi:10.1007/s00360-015-0929-1) contains supplementary material, which is available to authorized users.

## Introduction

Torpor is a state of metabolic depression that reduces energy expenditure and water loss in cold and/or dry environments (Wyss 1932; Kayser 1961). In contrast to daily heterotherms that undergo short daily torpor bouts, hibernators remain torpid for days or even weeks, before they periodically rewarm to euthermic body temperature (*T*_b_) (Carey et al. 2003; Geiser 2013; Ruf and Geiser 2015). Prolonged torpor may also occur during summer, in which case it is called summer dormancy or estivation (e.g. Macmillen 1965; Bieber and Ruf 2009). Although torpor is widespread among mammals and birds and occurs from the Arctic to the tropics (reviewed in Dausmann et al. 2004; Geiser and Körtner 2010; Ruf and Geiser 2015), the impact of torpor on life-history traits is not fully understood. There is increasing evidence that, apart from minimizing energy expenditure, the avoidance of predation—because hibernators remain motionless and well hidden in underground burrows—is another selective advantage of extended torpor (Bieber and Ruf 2009; Turbill et al. 2011; Geiser and Brigham 2012; Bieber et al. 2014). Generally, hibernators have a high probability of surviving winter, with a 50 % higher annual survival probability compared with non-hibernators of similar body size (Turbill et al. 2011).

Dormice are arboreal, nocturnal rodents adapted to yearly fluctuations in seed production of European beech, *Fagus sylvatica*, a major food source for this species in the northern part of its range. In years of low beech seed abundance, dormice skip reproduction, presumably due to low probability of juvenile survival (Bieber 1998; Schlund et al. 2002; Ruf et al. 2006; Lebl et al. 2010). In years without reproduction, however, dormice are rarely recaptured yet exhibit high annual survival (Ruf et al. 2006; Lebl et al. 2011a). These findings could be explained by some members of the population using summer dormancy, in addition to winter hibernation, a phenomenon that has been observed under semi-natural conditions in enclosures (Bieber and Ruf 2009). However, since edible dormice do not eat during prolonged torpor, summer dormancy requires large energy reserves. Depositing large fat stores is enhanced by ad libitum feeding of dormice in outdoor enclosures (Bieber et al. 2014). Further, pygmy possums (*Cercartetus nanus*) fattened in the laboratory could hibernate for more than a year (Geiser 2007).

Consequently, summer dormancy or prolonged hibernation as reported by Geiser (2007) and Bieber and Ruf (2009) may have been triggered by the availability of surplus food. To date, there is no quantitative evidence that summer dormancy occurs in free-living dormice. Thus, the main purpose of our study was to determine whether it is used in the wild. To increase the probability of detecting summer dormancy in addition to hibernation, we intentionally used first captured animals in a non-reproductive year, triggered by the absence of beech seed production (2012).

## Materials and methods

### Study site

The field study was conducted in Lower Austria near Vienna between June 2012 and August 2014. All edible dormice were free living in the Vienna Woods, Austria (48°05′N, 15°54′E; altitude 400–600 m a.s.l.). This area included approximately 650 ha of deciduous forest dominated by beech (*Fagus sylvatica*, 60 %) and spruce (*Picea abies*, 15 %), with oak (*Quercus robur*) occurring only rarely [<1 %; for further information about the study site, see Lebl et al. (2011b)]. We used two data loggers (EL-USB-2, Lascar Electronics, Whiteparish, UK, accuracy: ±0.5 °C) installed in shaded spots 2 m above ground to measure ambient temperature [*T*_a_; mean *T*_a_ between 1 June 2012 and 30 June 2013: 8.6 °C, maximum summer *T*_a_ (day): 33.5 °C, minimum winter *T*_*a*_ (night): −13 °C]. Soil temperature (used as a proxy for burrow temperature during hibernation) was measured with iButtons (DS1922L, Maxim, Dallas, USA; accuracy: ±0.5 °C), buried 60 cm below ground at the two air *T*_a_ sites (yearly mean soil temperature: 9.6 °C, maximum summer temperature: 28.2 °C, minimum winter temperature: 1.1 °C). The beech mast pattern observed at our study site was the following: 2011—full mast, 2012—mast failure, 2013—full mast, 2014—mast failure.

### Capture and manipulation of animals

We checked 130 irregularly positioned nest boxes (height: 2–3 m) at fortnightly intervals (for further details about nest box distribution and position see Lebl et al. 2011b). Animals occupying the nest boxes (i.e. using them as a sleeping site or to rear their young) were captured during the active season (April–October). Newly captured individuals were sexed and marked with subcutaneous transponders (BackHome BioTec^®^,Virbac Limited, Bury St. Edmunds, UK; Tierchip Dasmann^®^, Greven, Germany). The animals were assigned to one of the three age classes (based on fur length and coloration; for details see Vietinghoff-Riesch 1960): Juveniles (before first hibernation), yearlings (after first hibernation) and adults (after second hibernation).

At the time of capture, the animals were weighed to the nearest 2 g using a 300 g spring balance (Pesola^®^, Baar, Switzerland). In dormice, variation in body mass is highly correlated with variation in white adipose tissue (WAT) depots (*r* = 0.84, *n* = 27; Schaefer et al. 1976). Notably, the slope of this relationship was close to 1 (0.98), indicating that variation in body mass directly reflects total body fat content.

We transported the 43 captured animals to the Department of Integrative Biology and Evolution in Vienna (distance ~50 km). They were housed alone in cages (59 × 49.5 × 41 cm) equipped with fresh branches and a small wooden nest box (provided with hay), animals had access to rodent chow (Altromin #7020, Altromin International, Lage, Germany) and water ad libitum. Additionally 25 g of apple was provided daily. Animals were housed under the natural light–dark cycle. After 2 days of acclimatization, animals were anaesthetized and wax-coated iButtons (DS1922L, Maxim, Dallas, USA; resolution 0.5 °C) were implanted intraperitoneally (*n* = 43) following a standard surgical procedure (for detailed description see Bieber et al. 2014). The same procedure was used for explantation (*n* = 17; after approximately 1 year, in one dormouse after approximately 2 years). Implantations were carried out in June and July 2012, logger removals between May 2013 and August 2014.

Only dormice weighing >80 g (i.e. yearling and adult dormice) were implanted with iButtons. After recovery (i.e. 7 days post surgery), animals were released into the nest box where they had been captured. The entrances to these nest boxes were closed between the time of capture and release to ensure that no other dormice occupied them.

### *T*_b_ recording

Data loggers were calibrated prior to implantation and re-calibrated after explantation in a water bath at five different temperatures (5, 10, 15, 20, and 40 °C) using a precision thermometer (Testo 950, Testo, Lenzkirchen, Germany, accuracy ±0.05 °C). The maximum deviation between actual and recorded temperature was 0.34 °C, which is below the resolution of iButtons.

**Fig. 1:**
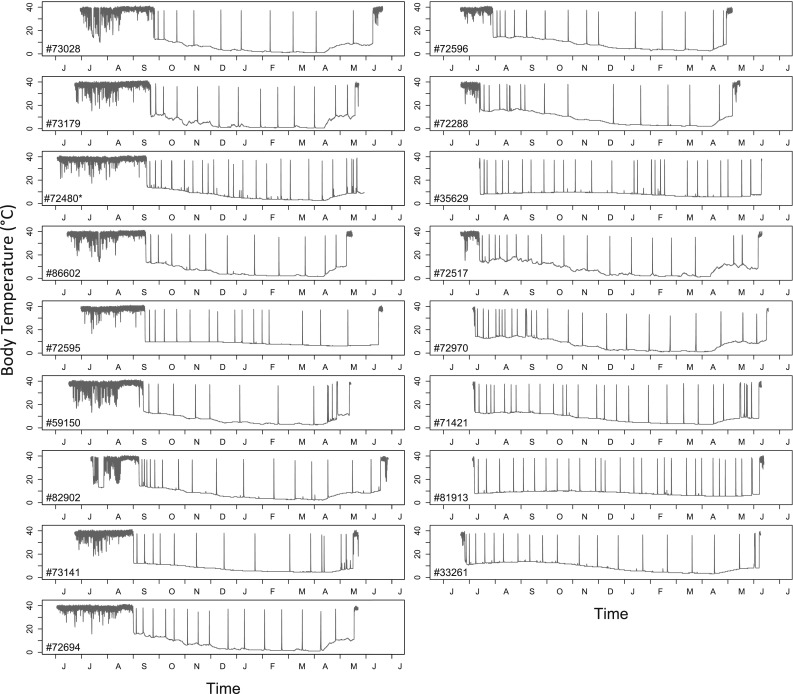
Year round records of core body temperature (*T*_b_) in free-living edible dormice in 2012/2013 (*n* = 17)

To enable a full year of data recording, body temperatures were recorded at 3850 s (~64 min) intervals, which correspond to a 1 year storage capacity of the loggers. To improve the temporal resolution of the duration estimates, all data were linearly interpolated (at six regularly spaced time points between actual measurements) prior to further analysis, resulting in time intervals of ~8 min. We used a threshold temperature of 25 °C to define the onset and termination of torpor bouts during prolonged torpor. Because summer dormancy blended into hibernation without disruption (Fig. 1), we henceforth simply use the term “hibernation” for prolonged (>7 days) multiday torpor, irrespective of season. Hibernation onset was defined as the first time-point after which *T*_b_ remained below 25 °C for at least 24 h. Brief periods (2–7 days) of multiday torpor that occurred during summer in three individuals were not, however, classified as hibernation onset because they were not followed by a regular hibernation season, which usually last >7 months. Hibernation was considered to end when *T*_b_ remained >25 °C for more than 72 h. Termination of hibernation and hence duration of the hibernation season could not be determined for one animal due to an iButton running out of memory while the animal was still hibernating. Additionally, we computed the number of arousals (number of phases with *T*_b_ > 25 °C), as well as the duration of interbout euthermic phases (IBE; hours *T*_b_ > 25 °C) and each torpor bout duration (hours *T*_b_ < 25 °C) (Bieber et al. 2014).

Dormice also exhibited short, shallow torpor bouts mainly on a daily basis, predominantly during the first part of the active season (Fig. 1). To determine the onset, endpoint and duration of these bouts, we used a threshold *T*_b_ of 32 °C to allow comparisons with prior studies on this species (Wilz and Heldmaier 2000). For the period prior to hibernation onset, we computed mean daily minimum *T*_b_ as an index for overall torpor use, as well as mean daily maximum *T*_b_. These analyses were restricted to dormice (*n* = 12) that remained active for at least 2 weeks after being released.

### Statistical analyses

All statistical tests were carried out using R 3.0.2 (R Development Core Team 2013). We used a Shapiro-Wilks test to assess the normality of model residuals.

We used linear models, followed by type III sum-of-squares ANOVA (using library ‘car’; Fox and Weisberg 2011) to assess variation between individuals in hibernation characteristics. We report *F* and corresponding *P* values from models that minimized Akaike’s Information Criterion (AIC). Since our sample size was 17 animals with temperature loggers recaptured, we restricted predictor variables to main effects of spring body mass prior to hibernation, age-class and sex, based on previous findings (Bieber et al. 2014).

## Results

### Timing of hibernation and hibernation patterns

**Fig. 2:**
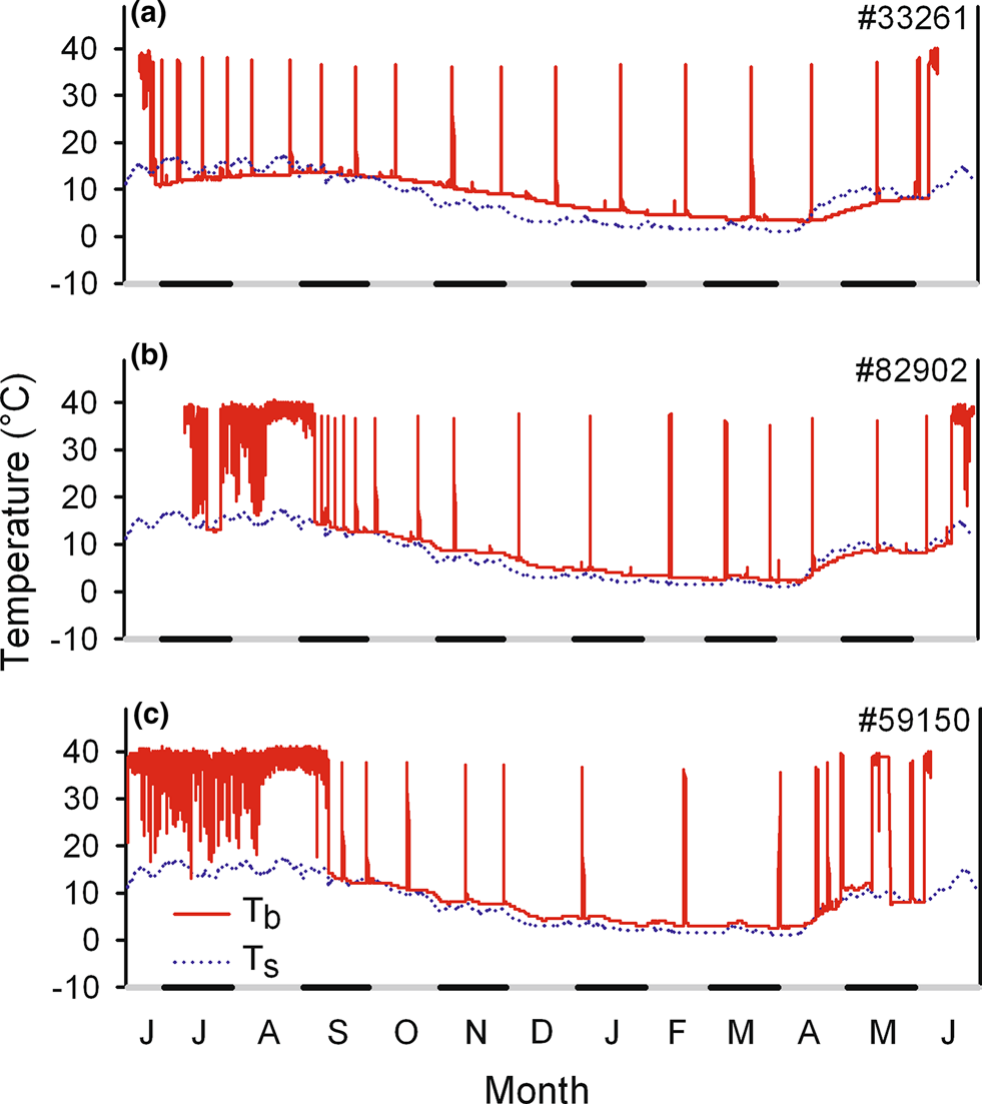
Year round records of core body temperature (*T*_b_ *solid lines*) in three free-living edible dormice in 2012/2013. **a** Example of an animal showing an early onset of hibernation in June and staying in hibernation for 331 days. **b** An animal using multiday torpor during the active season in July. **c** An individual showing a long active season, entering hibernation in September. Torpor *T*_b_ during hibernation and summer dormancy was close to soil temperature (*T*_s_ *dotted lines*)

Core body temperature in free-living edible dormice indicated that the animals hibernated on average 9.4 ± 0.3 months (*n* = 17; Table S1, Fig. 1) in a year of beech seed failure in temperate Lower Austria. We recorded considerable variation in the duration of the hibernation season between individuals (7.8–11.4 months, Figs. 1, 2). Eight of 17 dormice began hibernation in June/July, with 5 of these achieving durations of hibernation seasons of 11 months or more (11.0–11.4 months, Table S1, example: Fig. 2a). Duration of hibernation season was positively correlated with the number of arousals from torpor (10–28; rho = 0.90, *P* < 0.001). Arousal rate (mean: 1.74 ± 0.1 month^−1^) was higher in animals with early hibernation onset (rho = 0.70, *P* < 0.01) and a long duration of the hibernation season (rho = −0.68, *P* < 0.01).

The maximum recorded duration of the hibernation season was 346 days (in an adult female, Fig. 2a). Duration of hibernation season was significantly related to spring body mass (range: 80–154 g; *F* = 11.4, *P* < 0.01) with heavier animals remaining in hibernation longest. However, after statistically adjusting for differences in body mass, yearlings showed relatively longer durations of hibernation seasons than adults (*F* = 56.8, *P* = 0.02). As expected, duration of hibernation season was negatively correlated with hibernation onset (*r* = −0.92, *P* < 0.001).

**Fig. 3:**
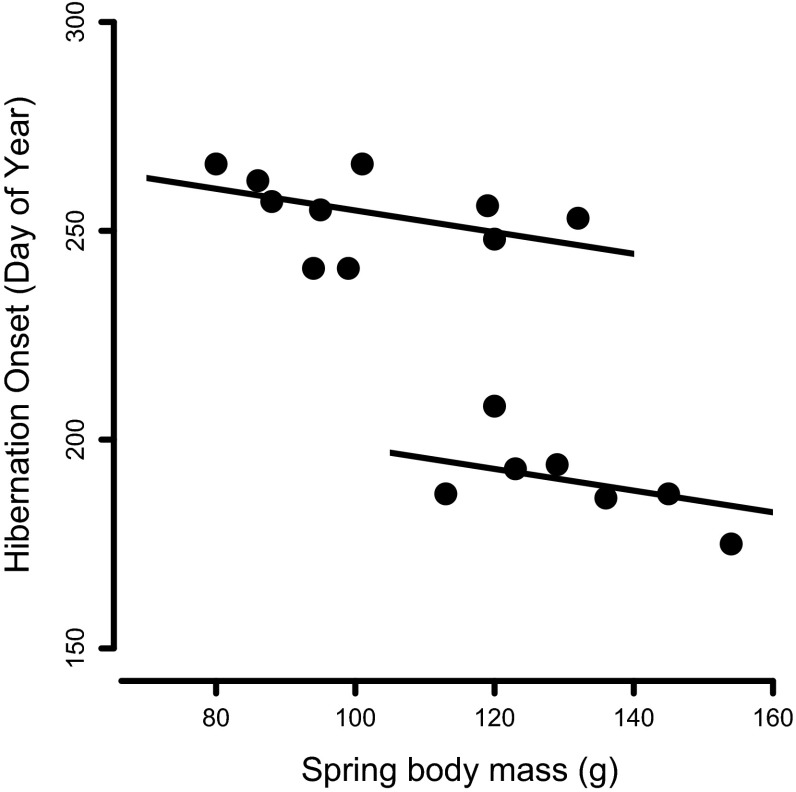
Hibernation onset as a function of body mass in late spring (May–early July; *n* = 17). The onset of hibernation had a bimodal distribution. Hibernation onset tended to be early in dormice with high spring body mass. The intercepts of the two regression lines shown were significantly different (*F* = 56.7, *P* < 0.001), while—within groups—the effect of body mass on hibernation onset was not significant (*F* = 3.3, *P* = 0.09)

Spring body mass was the only significant predictor for the time of hibernation onset (*F* = 20.4, *P* < 0.01), and large body mass was associated with early hibernation onset. However, the distribution of hibernation onset dates was bimodal (Fig. 3), with prolonged hibernation starting either early (≤day of year 208, i.e. July 28) or late (≥241, i.e., August 30). An ANCOVA with separate regressions through these subgroups had a much lower AIC (76.7) and explained more variance (adjusted *R*^2^ = 0.93) than a single regression (AIC = 108.2, *R*^2^ = 0.55). The regression slopes for the two subgroups were not significantly different (*F* = 0.99, *P* = 0.33, Fig. 3). Within the two groups, the effect of body mass on hibernation onset was also not significant (*F* = 3.3, *P* = 0.09). However, the intercepts of the two regression lines were significantly different (*F* = 56.7, *P* < 0.001), indicating that dormice that entered hibernation early had a higher mean body mass.

Mean torpor bout duration was 417.1 ± 21.6 h (*n* = 16, mean minimum: 94.2 ± 18.1 h; mean maximum: 832.2 ± 59.4 h), and mean IBE duration was 5.3 ± 0.2 h (*n* = 16, mean minimum: 3.3 ± 0.1 h, mean maximum: 10.0 ± 1.5 h). There were no significant effects of body mass, age-class or sex on torpor bout or IBE durations. Mean torpor bout duration was shorter, however, the earlier the onset of the hibernation season (*F* = 7.0, *P* = 0.02).

### Short torpor and pre-hibernation period

**Fig. 4:**
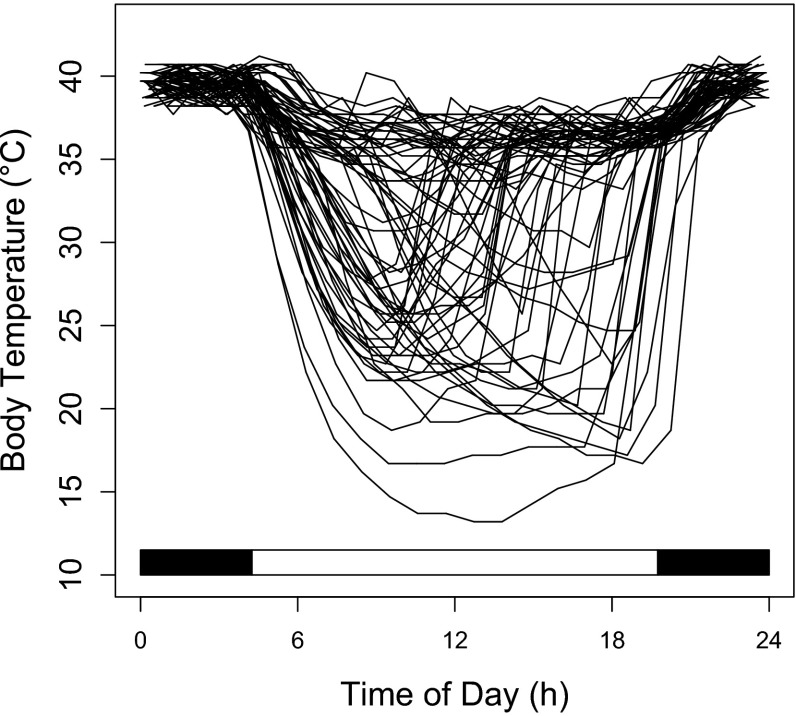
Temporal pattern of *T*_b_ during summer in an edible dormouse (# 59150; cf., Fig. 2) as a function of time of day during the summer-active season (mid June—early September).* Each line* shows a 24-h trace of *T*_b_. On many days, the animal exhibited short torpor bouts (when *T*_b_ dropped below 32 °C). The *black bars* on the abscissa indicate scotophase during the middle of the recording period

During the active season (April to October), most dormice used short shallow torpor bouts (Figs. 1, 2c), which occurred predominantly in their resting phase during the day (Fig. 4). Mean torpor bout duration was 7.44 ± 0.90 h, the mean time of entrance (*T*_b_ < 32 °C) was at 08:02 ± 0.32 h, mean time of return to euthermia (*T*_*b*_ > 32 °C) at 14:44 h ± 0.31 h. Within individuals, the timing of entry into torpor was less variable than arousal from torpor (Fig. 4). Short torpor bouts were not entirely restricted to daylight hours, as three dormice also showed multiday torpor with durations of up to 161 h (6.7 days; Fig. 1, 2b). The low and stable *T*_b_ during these rare multiday torpor events (similar to soil temperature, Fig. 2b) suggests that these phases were, as during hibernation, spent in underground burrows.

**Fig. 5:**
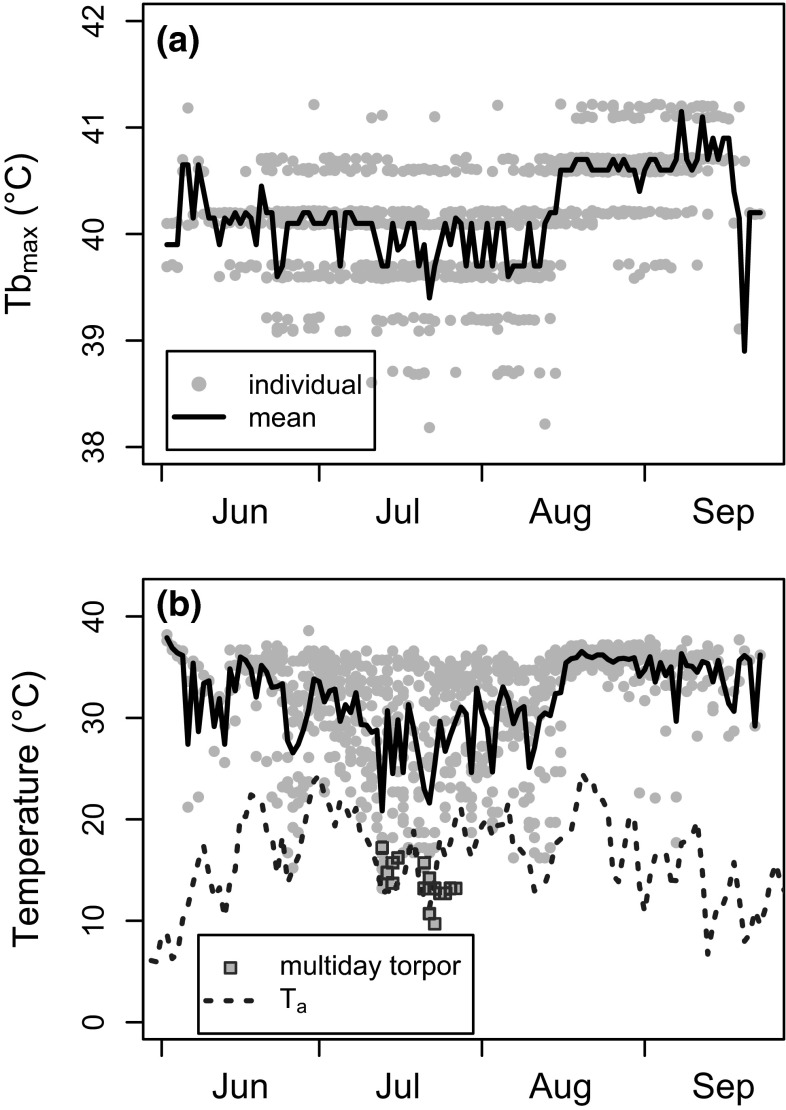
**a** Mean daily maximum *T*_b_ (*line*) and individual daily maximum *T*_b_ (*grey dots*) between release and hibernation onset among dormice (*n* = 12) that remained active for at least 2 weeks. The horizontal bands of *grey dots* result from the limited resolution of the iButtons. Nocturnal maximum *T*_b_ notably increased in August and September. **b** Mean daily minimum *T*_b_ (including bouts of torpor; *solid line*) and individual daily minimum *T*_b_ (*grey dots/squares*) between release and hibernation onset among dormice (*n* = 12) that remained active for at least 2 weeks. The *dashed line* shows air temperature at the study site; the *squares* indicate individual daily minimum *T*_b_ during multiday torpor events. Note that minimum *T*_b_ during multiday torpor was frequently well below *T*_a_ (up to ~6 °C), indicating that these episodes likely occurred in underground burrows

Most of the dormice that began hibernating late in the season (August to September, *n* = 12) reduced their use of short shallow torpor bouts in the last weeks prior to hibernation (Figs. 1, 5). During this period, daily maximum *T*_b_, which always occurred at night, increased to ~40.5 °C (Fig. 5a). There was no correlation between mean minimum daily *T*_b_ (*n* = 12 animals), as an integrative measure of torpor use, and mean daily *T*_a_ (*r* = 0.11, *t* = 1.21, *df* = 112, *P* = 0.228, Fig. 5b).

## Discussion

A duration of the hibernation season of up to 11.4 months has never been detected in any other free-living endotherm previously. It was known that edible dormice have the capacity to undergo summer dormancy and can massively extend the hibernation period under semi-natural conditions (Bieber and Ruf 2009). However, our current data show that not only the maximum duration but also the average duration of the hibernation season of 9.4 months in free-living dormice is approximately 1 month longer than described previously (Bieber et al. 2014).

Comparably extreme cases of prolonged hibernation were only observed under laboratory conditions at constant ambient temperature in marsupial pygmy possums that had been substantially fattened by providing food ad libitum (Geiser 2007). Even species living in extremely harsh environments, like the Arctic, are not known to hibernate for such extended periods (Barnes 1996). The large individual variation in duration of the hibernation season observed here (7.8–11.4 months) within the same population indicates that dormancy is not solely determined by environmental conditions, such as climate or food availability. This is consistent with the hypothesis that hibernation serves other functions in addition to lowering energy expenditure (Kobbe et al. 2011; Geiser and Brigham 2012; Ruf et al. 2012). In edible dormice, but possibly also in other small hibernators, avoiding predation seems to be a major selective advantage (Bieber and Ruf 2009; Turbill et al. 2011; Bieber et al. 2014). However, only animals with high body mass (i.e. high body fat reserves; Schaefer et al. 1976) started to hibernate early. Most likely large energy reserves are an important prerequisite to maximize the use of hibernation. However, our current data demonstrate that even under natural conditions, a substantial fraction of dormice can obtain sufficient energy reserves (i.e. body fat) to reduce the annual active season to less than a month.

Although energy-rich food seems crucial for reproduction in dormice (Fietz et al. 2004, 2005; Kager and Fietz 2009; Lebl et al. 2010), it is important to note that summer-active dormice are able to gain mass and deposit fat stores from alternative food resources (leaves, fruits), even in years of mast failure (Bieber 1998) such as 2012 at our study site. The need for continued food intake depends on body fat reserves immediately after emergence from hibernation. It is known that dormice carry surplus body fat reserves from autumn into the next active season: Heavier animals prior to hibernation are also heavier after hibernation (Bieber et al. 2014). This is probably an important factor because mast failure years, such as the first year of our study, are typically preceded by full beech masting years, which provide abundant energy-rich food and allow extreme fattening. Due to small-scale differences in local food abundance (Lebl et al. 2011b), and possibly the quality of hibernacula, there still will be variation among dormice in spring body masses after hibernation as observed here.

The fact that animals showed a variable number of arousals during hibernation (10–28, Fig. 1) can be largely attributed to the simple fact that a long duration of the hibernation season gives more time for rewarming phases. Additionally, the high frequencies of arousal were also caused by high burrow *T*_a_ in those animals that started to hibernate in summer (Figs. 1, 2a). It is well known that elevated burrow *T*_a_ and consequently higher minimum *T*_b_ cause shorter torpor bouts and thus more frequent arousals during hibernation (Fig. 2c; see also Geiser and Kenagy 1988; Geiser and Broome 1993; Bieber and Ruf 2009). On the other hand, rewarming from higher *T*_b_ will reduce arousal costs (Song et al. 2000) and may attenuate possible cellular damage, which seems to be associated with large fluctuations in *T*_b_ and mitochondrial respiration (Carey et al. 2003). Still, due to increased arousal frequency and higher TMR (Song et al. 2000) hibernation at higher burrow *T*_a_ is probably more energetically costly than at lower *T*_a_, because rewarming episodes cause >70 % of the energy expenditure during hibernation (Tucker 1965; Wang 1985). Early onset of hibernation when soil temperatures are still high (Fig. 2a) therefore most likely requires higher energy reserves, which partly explains why only heavier individuals tended to do this (Fig. 3) and hibernated longer. Even these individuals, however, appeared to seek out relatively cool and thermally buffered hibernacula most likely to reduce arousal frequency. Presumably, these animals hibernated at least 60 cm below the surface as their *T*_b_ in summer commonly fell below the soil temperature at this depth (Fig. 2a).

Interestingly, hibernation onset did not occur continuously over the active season (Fig. 3). Instead, dormice started to hibernate either early (06–28 Jul) or late (30 Aug–24 Sep), which suggests two separate time windows for hibernation onset. This raises questions about the underlying internal clock mechanisms that generate these windows. Dormice are known to have somewhat unusual circannual rhythms that can be entrained by both photoperiod (Morrison 1964) and ambient temperature (Jallageas et al. 1989). In addition, there is evidence for infradian rhythms in this species with a period of approximately 2 months (Mrosovsky et al. 1980). All of these characteristics may be involved in the bimodal timing of hibernation onset, but clarifying the mechanism behind this requires more research. Functionally, our results suggest that in non-reproductive years, there are two alternative ways in which dormice can spend the summer season: either rapidly return to hibernation or stay active and forage until the fall. Body condition, which may be affected by food abundance and reproductive effort in the previous year as well as the quality of underground hibernacula, appears to have some effect on the decision for which route to take. However, the overlap in spring body mass between both groups of dormice (Fig. 3) indicates that there must be other factors involved. These factors may include food availability and quality within the home range of individuals after their emergence from the previous hibernation season or even local predation pressure.

We acknowledge that extremely early onsets of hibernation may have been caused by subjecting the animals to surgery and/or by the implanted temperature loggers. There are, however, three reasons that refute this interpretation: (1) summer dormancy was previously observed in edible dormice under semi-natural conditions in animals not implanted with loggers (Bieber and Ruf 2009); (2) all animals we studied were only released in good health after full recovery and (3) effects of surgery or logger implantation do not explain why hibernation onset was significantly associated with body mass, with the earliest onsets occurring in dormice in good condition (Fig. 3). Notably, the yearly recapture rate of animals implanted with loggers (17 of 43 = 40 %) was actually slightly higher than that of non-implanted yearling and adult dormice during the same time interval (32 %; 163 animals captured in 2012 and 52 recaptured in 2013 at the same study site). Hence, there was no evidence for adverse effects of logger implantation on survival rates.

Dormice that did not start to hibernate until fall frequently used short torpor bouts during the diurnal resting phase, particularly in the first part of the active season (Figs. 1, 5). Although shallow torpor is known to substantially lower costs for thermoregulation (Wilz and Heldmaier 2000; Fietz et al. 2004), mean minimum daily *T*_b_ was not correlated with *T*_a_ (Fig. 2a, b, Fig. 5). Hence, it seems that short torpor bouts (<24 h) were not triggered by low *T*_a_. Short torpor bouts were not accompanied by a retreat into underground burrows, as we commonly found animals torpid and cold to the touch in above-ground nest boxes during the day. However, three animals also used multiday torpor in summer (e.g. Fig. 2b) which may have been expressed underground. These multiday torpor events occurred only in July, when *T*_a_ was unseasonably low (Fig. 5b) and precipitation high. This leads us to conclude that dormice use multiday torpor in summer when unfavourable weather conditions preclude foraging by temporarily retreating into underground burrows, at least in mast failure years.

In contrast to some other hibernators that show short bouts of torpor just prior to hibernation onset (Sheriff et al. 2012), short episodes of torpor in dormice largely ceased to occur in the second half of the active season (Figs. 1, 2b, c). This was accompanied by a rise in nocturnal maximum *T*_b_, starting in mid-August (Fig. 5a). The high *T*_b_ late in the active season was most likely the result of increased foraging efforts, that is, increased locomotor activity, and heat increment of feeding. While it is known that rodents in hot environments have to cope with increasing *T*_b_ (Chappell and Bartholomew 1981; Bondarenco et al. 2014), we are not aware of data showing *T*_b_ reaching >40 °C during activity in animals without heat load in a temperate zone forest. The fact that this phenomenon occurs in edible dormice may be related to both their arboreal lifestyle, which entails relatively high costs of locomotion (Karasov 1992), and their need to gain body fat reserves for hibernation. Mid-August to early October is when dormice rapidly gain pre-hibernation mass (Fietz et al. 2005; Lebl et al. 2010). Increased foraging may also be the underlying reason why the use of torpor was reduced at this time (Fig. 1). As suggested previously (Ruf and Heldmaier 1992; Nowack et al. 2013) torpor may have adverse effects on energy intake because low *T*_b_ significantly decreases rates of nutrient absorption in the digestive tract (Carey 1989).

## Conclusions

Our results demonstrate that previous observations of extensive use of prolonged torpor, including dormancy during summer, were not caused by ad libitum feeding in edible dormice under laboratory conditions but are part of the natural physiological and behavioural repertoire of this species. However, prolonged hibernation is only one possible strategy employed in non-reproductive years by a fraction of animals, probably to maximize predator avoidance. An alternative tactic is a combination of short torpor, largely diurnal, combined with continuous nocturnal foraging during the active season. The extreme use of hibernation used by some individuals is clearly linked to forgoing reproduction in *Glis glis*. However, with more studies of heterothermia in free-living animals, the general pattern seems to be for more extensive use of torpor than predicted from laboratory settings (Ruf and Geiser 2015). We would not be surprised if almost year-round hibernation will also be found in free-living individuals of other species.

## Electronic supplementary material

Supplementary material 1 (DOCX 22 kb)
