# Supplementary material for: How to spend the summer? Free-living dormice (Glis glis) can hibernate for 11 months in non-reproductive years
Source: J Comp Physiol B. 2015 Aug 21;185(8):931–9. doi: 10.1007/s00360-015-0929-1 (PMC4628641; doi:10.1007/s00360-015-0929-1)
Supplement: Supplementary file 1 — Supplementary material 1 (DOCX 22 kb) [file 360_2015_929_MOESM1_ESM.docx]

**Supplemental material**

Table S1, related to Figure 1: Hibernation characteristics and initial body masses of all recaptured dormice (a = adult, y = yearling; f = female, m = male).

| **ID** | **sex** | **age** | **Date of hibernation  start** | **Date of hibernation  end** | **hibernation duration (months)** | **number of arousals (while hibernating)** | **body mass at implantation [g]** |
| --- | --- | --- | --- | --- | --- | --- | --- |
| 73028 | f | y | 09/24/2012 | 06/08/2013 | 8.43 | 10 | 80 |
| 72596 | m | a | 07/28/2012 | 04/29/2013 | 9.01 | 14 | 120 |
| 73179 | m | y | 09/20/2012 | 05/18/2013 | 7.89 | 12 | 86 |
| 72288 | m | a | 07/13/2012 | 05/06/2013 | 9.74 | 15 | 123 |
| 72480 | f | y | 09/15/2012 | N/A | N/A | N/A | 88 |
| 35629 | f | a | 07/14/2012 | 06/10/2013 | 11.10 | 26 | 129 |
| 86602 | m | a | 09/14/2012 | 05/08/2013 | 7.75 | 11 | 119 |
| 72517 | m | y | 09/24/2012 | 06/08/2013 | 8.43 | 17 | 101 |
| 72595 | f | y | 09/13/2012 | 06/15/2013 | 9.02 | 15 | 95 |
| 72970 | f | y | 07/07/2012 | 06/15/2013 | 11.26 | 24 | 113 |
| 59150 | m | a | 09/11/2012 | 05/11/2013 | 7.96 | 12 | 132 |
| 71421 | f | a | 07/07/2012 | 06/06/2013 | 10.96 | 28 | 145 |
| 82902 | f | a | 09/06/2012 | 06/17/2013 | 9.33 | 16 | 120 |
| 81913 | f | a | 07/06/2012 | 06/07/2013 | 11.02 | 28 | 136 |
| 73141 | m | y | 08/30/2012 | 05/17/2013 | 8.51 | 15 | 99 |
| 33261 | f | a | 06/25/2012 | 06/06/2013 | 11.37 | 19 | 154 |
| 72694 | m | a | 08/30/2012 | 05/17/2013 | 8.53 | 15 | 94 |
| mean |  |  | 08/12/2012 | 05/27/2013 | 9.39 ± 0.33 | 17.31 ± 1.49 | 113.8 ± 5.27 |
